# Moving Stimuli Are Less Effectively Masked Using Traditional Continuous Flash Suppression (CFS) Compared to a Moving Mondrian Mask (MMM): A Test Case for Feature-Selective Suppression and Retinotopic Adaptation

**DOI:** 10.1371/journal.pone.0098298

**Published:** 2014-05-30

**Authors:** Pieter Moors, Johan Wagemans, Lee de-Wit

**Affiliations:** Laboratory of Experimental Psychology, University of Leuven (KU Leuven), Leuven, Belgium; University of Minnesota, United States of America

## Abstract

Continuous flash suppression (CFS) is a powerful interocular suppression technique, which is often described as an effective means to reliably suppress stimuli from visual awareness. Suppression through CFS has been assumed to depend upon a reduction in (retinotopically specific) neural adaptation caused by the continual updating of the contents of the visual input to one eye. In this study, we started from the observation that suppressing a moving stimulus through CFS appeared to be more effective when using a mask that was actually more prone to retinotopically specific neural adaptation, but in which the properties of the mask were more similar to those of the to-be-suppressed stimulus. In two experiments, we find that using a moving Mondrian mask (i.e., one that includes motion) is more effective in suppressing a moving stimulus than a regular CFS mask. The observed pattern of results cannot be explained by a simple simulation that computes the degree of retinotopically specific neural adaptation over time, suggesting that this kind of neural adaptation does not play a large role in predicting the differences between conditions in this context. We also find some evidence consistent with the idea that the most effective CFS mask is the one that matches the properties (speed) of the suppressed stimulus. These results question the general importance of retinotopically specific neural adaptation in CFS, and potentially help to explain an implicit trend in the literature to adapt one’s CFS mask to match one’s to-be-suppressed stimuli. Finally, the results should help to guide the methodological development of future research where continuous suppression of moving stimuli is desired.

## Introduction

Since Crick and Koch [1] set out a framework for studying the neural correlates of consciousness, a number of paradigms have been developed to study the neural activity associated with purely perceptual changes that allow one to study the correlates of consciousness without changing the visual input. Continuous flash suppression (CFS) is a psychophysical technique that enables this by suppressing stimuli much more reliably than in standard binocular rivalry paradigms, and with much longer presentation times possible compared to visual masking paradigms [2]. In essence, CFS is highly similar to binocular rivalry: Two different images are presented to the same regions of both eyes, but in one eye, a rapidly changing stimulus is presented, which effectively suppresses the stimulus in the other eye for relatively long periods of time (i.e., units of seconds rather than seconds, [2]). Traditionally, this changing stimulus is a Mondrian fashioned pattern of rectangles and squares of random size and color that changes every 100 ms (10 Hz).

Since CFS was introduced as a technique to reliably suppress stimuli from visual awareness, it has been used in more than 100 studies. In these experiments, CFS has been applied in two different ways. First, it has been used to present stimuli in the absence of awareness and to study the influence of the presentation of these subliminal stimuli on a subsequent task with visible stimuli. For example, Jiang, Costello, Fang, Huang, and He [3] report an attentional effect of unconsciously presenting erotic pictures. CFS has been used in this way to study the orientation aftereffect [4], [5], motion aftereffect [6], [7], simultaneous motion contrast [8], face adaptation [9]–[11], as well as a preconscious attentional bias in cigarette smokers [12].

Secondly, CFS has been most often put into practice in the so-called “breaking CFS paradigm” (a term coined by Stein, Hebart, & Sterzer [13], based on the paradigm introduced by Jiang, Costello, & He [14]). In this paradigm, participants have to detect when a stimulus suppressed through CFS breaks through the mask. Differential breakthrough times for different conditions are then taken as evidence that some kind of unconscious representation of the different stimuli must have been generated in order for them to break through at differential rates. Using this technique (abbreviated as “b-CFS”), Jiang et al. [14] showed that faces break through suppression faster than inverted faces. Since b-CFS was introduced, it has been used widely. Costello, Jiang, Baartman, McGlennen, and He [15] provided evidence for unconscious semantic word priming, Bahrami, Vetter, Spolaore, Pagano, Butterworth, and Rees [16] for unconscious numerical processing, Xu, Zhang, and Geng [17] for gaze cuing in the absence for awareness, Wang, Weng, and He [18] for perceptual grouping of a Kanizsa triangle under CFS and Mudrik, Breska, Lamy, and Deouell [19] documented that incongruent scenes break through faster than congruent scenes.

Despite the broad and increasing employment of this method, it is still is not clear which factors contribute to the effectiveness of CFS in suppressing stimuli from awareness. Some authors imply that the effectiveness of CFS derives from its saliency. For example, Bahrami, Lavie, and Rees [20] describe their CFS mask as “highly salient, high-contrast, and rapidly changing blue masks” (p. 510); Raio, Carmel, Carrasco, and Phelps [21] refer to a “salient dynamic stimulation” (p. R477). Other authors [22]–[28] describe their CFS masks similarly. The most widespread explanation for the effectiveness of CFS, however, has been a general reduction in neural adaptation due to the fast transients associated with the mask (as in [29], [30]). That is, the input at a (retinotopic) location is updated every ∼100 ms, causing neurons with a receptive field at that location to show less neural adaptation compared to static input. Indeed Tsuchiya et al. [29] say: “We imagine that the enduring effectivess of CFS arises from its relative immunity to adaptation owing to the repeated presentation of a new stimulus” (p. 1075). Yang and Blake [30] are more explicit and articulate: “Perhaps, then, the rapid, repetitive changes in the successively presented, random configurations of a CFS display minimize its tendency to undergo neural adaptation (…)” (p. 11). Thus, because at every retinotopic location features such as orientation and contrast change, neurons responsive for these features tend to adapt less compared to static input. In this sense CFS can be understood as a form of binocular rivalry, in which percept switches have been explained (in part) as the result of neural adaptation to the dominant percept and competition between monocular neurons in low-level visual areas [31], [32]. Since the interocular competition process in binocular rivalry has mostly been characterized as happening in low-level visual areas (although recent models acknowledge the possibility for competition between different levels in the hierarchy of the visual system [33], [34]), we focus on the extent to which retinotopically specific neural adaptation can help to predict the effectiveness of CFS (see the General Discussion for further discussion of the role of higher-order adaptation processes in CFS). Framing the effectiveness of CFS as preventing retinotopically specific neural adaptation due to these fast changes in the mask would imply that the more changes over time the mask contains, the more effective CFS should be Indeed, this assumption also appears to be implicit in the literature when the refresh rate of the CFS mask is changed. Although most authors continue to use the traditional 10 Hz refresh rate as suggested in [2], when they do not, the refresh rate is mostly increased. Of the 81 studies we considered, 72% used the canonical 10 Hz refresh rate and 20% employed a refresh rate of more than 10 Hz. Indeed Xu et al. [17] increased the refresh rate with the explicit assumption that this would lead to more robust interocular suppression than the traditional 10 Hz frequency.

This explanation in terms of a reduction to retinotopically specific neural adaptation provides no immediate explanation for the way in which the traditional Mondrian mask is often adapted in the literature when masking specific stimuli in different studies. Different authors seem to adapt the characteristics of the CFS mask to match the characteristics of the to-be-suppressed stimulus. We provide three illustrative examples. First, Stein et al. [13] note that, although regular CFS allows for masking faces, it is much more effective to mask faces with a mask consisting of ellipses instead of squares. Second, the study of Bahrami et al. [20] used random geometrical shapes, contours and moving dots to suppress line drawings. Again, this adapted mask appears more similar to the to-be-suppressed line drawings than the standard Mondrian mask. Third, Sweeny, Grabowecky, and Suzuki [35] used a mask of randomly generated non-filled ellipses to mask an open or closed curve.

Only recently the importance of the characteristics present in the CFS mask has been highlighted as an important factor with respect to the effectiveness of CFS [30], [36], [37]. Indeed, Tsuchiya and Koch [2] never explicitly motivated their choice for the rapidly changing and flickering Mondrian-style rectangles as an effective CFS mask. Yang and Blake [30] proposed to address this issue by studying the effectiveness of CFS in relation to the properties of the mask and the suppressed stimulus. With respect to the spatial properties of the mask and suppressed stimulus, their results show that (1) it is harder to mask stimuli with high spatial frequency properties and (2) that stimuli with low spatial frequency properties are most effectively masked with CFS masks containing mostly low spatial frequencies.

Along the same line, an earlier study by Maehara et al. [37] reported almost no difference between suppression strength of a static and a flickering mask in suppressing a target stimulus when the spatial frequencies of mask and target were at least 1.6 octaves away from each other. Based on this result, Maehara et al. [37] proposed that the effectiveness of CFS presumably stems from within-channel masking.

Thus, it seems to be the case that the depth of suppression during CFS is not fixed, but rather depends on the interaction between the characteristics of the mask with the suppressed stimulus. This perhaps reintegrates our understanding of CFS with existing studies of binocular rivalry in general concerning feature-selectivity and the effect of shared stimulus complexity on suppression strength (e.g., [38]–[40]).

The potential importance of feature-selective competition in CFS does not rule out a role for adaptation-based explanation of its effectiveness. Indeed, for all these examples it is hard to disentangle the contribution of retinotopic neural adaptation and feature selectivity. In the present study we explore the relative contributions of retinotopic adaptation and feature competition by manipulating the properties of the CFS mask in such a way that feature overlap with the suppressed stimulus and retinotopic adaptation can be disentangled.

### The Present Study

As already highlighted, CFS is commonly described as a highly effective technique to suppress stimuli from visual awareness reliably and for longer time periods [2], [29]. Since CFS potentially offers long suppression times, it provides an excellent opportunity to be used in the context of suppressing dynamic stimuli that change over time (e.g., random-dot motion, biological motion, etc.). During pilot testing, however, we observed that regular CFS did not provide an effective means of suppressing moving stimuli. Rather, it appeared that introducing spatiotemporal continuity (e.g., motion) into our CFS-style mask seemed to be required to provide useful suppression times. The need to introduce continuous spatiotemporal signals into the mask does not seem to logically follow from what would be predicted from an account based on a reduction to retinotopically based neural adaptation in driving the effectiveness of CFS. Indeed, the spatiotemporal continuity we used to develop effective suppression should, if anything, be more prone to retinotopic neural adaptation than regular CFS. Given this observation, we set out to test whether a moving Mondrian mask (MMM) indeed provides a better means of suppressing a simple moving stimulus compared to regular CFS. In order to formally test the potential importance of retinotopic neural adaptation we explicitly operationalized the term and implemented a simple computation to quantify the degree of retinotopically specific neural adaptation (see Methods & Results of Experiment 1). Thus, the goal of this study was to show that, for moving stimuli, a release from retinotopically specific neural adaptation due to the changes in the mask over time is not the only mechanism that drives the effectiveness of CFS nor is it potentially the most dominant or important in a given context [30], [37].

To preview our results, our formalization of retinotopic neural adaptation proved to provide no basis for predicting the suppression strength of different MMMs containing different motion speeds.

## Experiment 1

In the first experiment, we used a MMM, manipulated the speed of the individual mask elements, and compared its effectiveness to a regular CFS mask in suppressing a moving target. Subsequently, we compared the observed effectiveness with what would be predicted to be the most effective mask based on a measure of retinotopically specific neural adaptation.

### Materials and Methods

#### Ethics statement

The study was conducted in line with the ethical principles regarding research with human participants as specified in The Code of Ethics of the World Medical Association (Declaration of Helsinki). The study was approved by the Ethical Committee of the Faculty of Psychology and Educational Sciences (EC FPPW) of the University of Leuven, and the participants gave written informed consent before starting the experiment.

#### Participants

Five students (1 male) of the undergraduate psychology program of the University of Leuven participated in the experiment for course credit. All had normal or corrected-to-normal vision. Every participant was unaware of the goal of the study.

#### Apparatus

Stimuli were shown on two 19.8-in. Sony Trinitron GDM F500-R (2048×1536 pixels at 60 Hz, for each) monitors driven by a DELL Precision T3400 computer with an Intel Core Quad CPU Q9300 2.5 GHz processor running on Windows XP. Binocular presentation was achieved by a custom made stereo set-up. Two CRT monitors, which stood opposite to each other (distance of 220 cm), projected to the left and right eye respectively via two mirrors placed at a distance of 110 cm from the screen. A head- and chin rest (15 cm from the mirrors) was used to stabilize fixation. The effective viewing distance was 125 cm. Stimulus presentation, timing and keyboard responses were controlled with custom software programmed in C# using Microsoft Visual Studio 2010.

#### Stimuli

**Figure 1:**
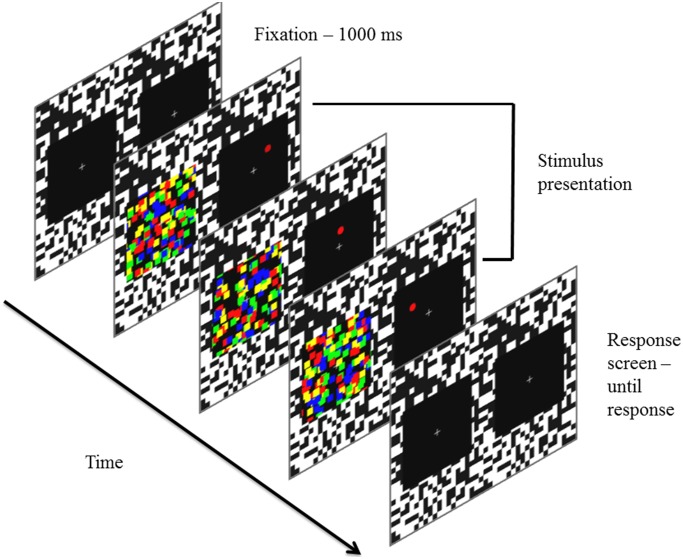
An example of the trial sequence.

A checkerboard pattern consisting of randomly positioned black and white squares of 0.37° by 0.37° was used to aid binocular fusion. The CFS masks consisted of 150 squares of equal size (0.46° by 0.46°). The color of the squares was either red, green, blue or yellow. The target stimulus was a red circle (diameter 0.46°). The target moved horizontally across a virtual square (5.5° by 5.5°) at a speed of 3°/s embedded in a larger square (7.32° by 7.32°). The mask was presented in the other eye within a bounding square of the same size as the larger square in the other eye (7.32° by 7.32°, see Figure 1).

The MMM differed from the traditional CFS mask. The main difference was that the individual elements of the mask were moving from frame to frame rather than flashing at randomly generated positions. Motion in the mask varied in six different directions (horizontal left/right and right/left, vertical up/down and down/up, diagonal bottom-left/top-right and top-left/bottom-right). Every mask element had one of these motion directions during the trial and the different motion directions were equally divided amongst the mask elements such that every motion direction was equally present in the display. For every motion direction, the colors of the individual elements were also evenly distributed. The initial position of every mask element was determined randomly with one constraint. To avoid that some parts of the display did not contain enough mask elements during a trial (creating blank spots), we divided the display into four quadrants and the positions, speeds and colors for each fourth of the mask elements were randomized within this quadrant. The size of the individual mask elements was the same as the size of the suppressed stimulus. When the positions of the mask elements overlapped, they were drawn on top of one another. Furthermore, when a mask element reached the edge of the display, it would reappear on the other side according to its motion trajectory.

#### Procedure

On each trial, the CFS mask was shown in the participant’s dominant eye. Eye dominance was determined prior to the start of the experiment with Porta’s test [41]. Consequently, the target stimulus was presented in the non-dominant eye. The target stimulus and the MMM/CFS mask would onset simultaneously, but the target began at a low contrast level, and faded in during the first 20 frames of the event. The target stimulus moved on a horizontal plane from the right side to the left and disappeared from the screen after 3.6 seconds. The target moved either above or below fixation at one of six motion paths (three above and three below fixation) randomly selected on every trial (but balanced across the motion conditions). These motion paths were equally spaced from each other. The distance between every of the three different motion paths was twice the target size. After the target disappeared from the screen, participants had to indicate if the target moved above or below the fixation cross. Contrast thresholds for the different mask speeds were determined by a one-up, two-down staircase procedure converging at 70.71% correct [42]. Two staircases were used for every mask speed. This resulted in twelve randomly interleaved staircases. The targets for the two staircases where given different starting values, in order to ensure the convergent consistency of the resulting thresholds. Because the task often was too easy for the high starting values, these staircases were accelerated by using a one-up, one-down procedure until the first incorrect response was recorded (for each of these staircases).

#### Design

Mask speed consisted of six different levels (1°/s, 2°/s, 3°/s, 5°/s, 8°/s and regular CFS) and two staircases were used for each mask speed. Participants performed 65 trials for each staircase, resulting in 780 (65 trials×6 speeds×2 staircases) trials in total. The number of trials per staircase was selected based on pilot testing. Staircases were randomly interleaved and participants had the opportunity to take small breaks in-between.

#### Simulations

Since we were interested in quantifying the degree of retinotopic neural adaptation for the different masks in our experiment, we conducted a simulation which implemented an approximation of the retinotopic representation of the input using Gabor filter banks often used in models of the early visual system (e.g., [43]). As highlighted above, the effectiveness of CFS has most often been explained as a reduction in neural adaptation due to the successive presentations of new random configurations. In order to provide a more explicit model of this how this adaptation process might work in early retinotopic areas, we convolved the stimuli in our experiment with a Gabor filter bank to extract orientation- and contrast-selective responses at each location of the image (akin to responses of neurons in primary visual cortex) and then used these responses as input to an adaptation simulation. An exponential decay function was used to represent adaptation to the input, and an exponential recovery function to represent the recovery of that retinotopic location when no input was present. These functions had the following form for decay and recovery, respectively:
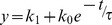


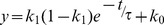
where k_0_ is the initial response level set at 1, k_1_ the asymptotic response level set at 0 for decay and 1 for recovery, and τ the time constant for the exponential. Note that this is not necessarily intended to provide a full or complete ‘model’ of retinotopic adaptation. Rather this simulation intends to make explicit what a simple approximation of retinotopic adaptation could look like. It is certainly possible that the adaptation dynamics in early retinotopic areas are much more complex, but this simulation enables us to quantify whether this very basic approximation of retinotopic adaptation can already predict our current results.

We simulated 999 trials of each condition and transformed each frame of the trial to a grayscale image. Next, we filtered each frame with two oriented (at 0 and 90 degrees) odd-symmetric Gabor filters with a spatial frequency set at the Nyquist frequency (412 c/image) and the standard deviation of the Gaussian set to:
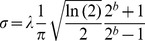
where λ equals the wavelength in pixels and b the bandwith in octaves. These settings were chosen to efficiently extract the responses to the edges of the different configurations in the CFS mask. The size of the filters was set to be four times the standard deviation of the Gaussian (varying filter size had no effect on the filter responses). This filtering procedure was implemented in the Python MDP package [44]. For each frame, this filtering step yields orientation- and contrast-specific responses for each pixel in the image, thresholded to be one out of five responses (2 orientations times 2 polarities and no response). Next, we calculated the degree of adaptation for each pixel by letting an “activation value” (starting at 1) decay with a time constant of four seconds as long as the input was present. When the input was no longer present, this “activation value” would recover again with a time constant of six seconds and this process would go on until the end of the trial. Note that this adaptation process was specific to one of the four possible filter responses. Both orientation and contrast polarity had to be the same across frames to yield adaptation. The time constants for adaptation and recovery were based on Giaschi, Douglas, Marlin, and Cynader [45]. This implementation yields an activity map for each location in the image for each of the four possible responses and we summed the values across all locations in an image to arrive at one summary statistic for the activation level associated with each mask condition. In other models of binocular rivalry, the output of the filtering step implemented here can be thought of as a representation of the ‘strength’ of the stimulus, which in these models is usually expressed as a single value. In most models of binocular rivalry the adaptation process is simulated on this one summary value of stimulus strength. In order to approximate the nature of adaptation on early retinotopic maps however we calculate a ‘stimulus strength’ value at every location (based on a Gabor filter), and apply adaptation at every location. In this way we try to isolate the contribution of ‘retinotopic’ adaptation to the effectiveness of CFS. The longer the extracted stimulus features remain the same at every retinotopic location, the more adaptation it will experience, thus the amount of adaptation at each location will be greater if the stimulus features remain the same over time. This should occur more in the slower motion conditions. The greater influence of adaptation in the slower motion conditions will result in a faster decrease in the ‘stimulus strength’ represented at every location, and this will be combined to influence the summary score. Thus, this summary score can be thought of as an (inverse) index of the degree of retinotopic adaptation, with more adaptation (associated with the slower motions) leading to a lower summary score.

### Results

Each threshold was determined by taking the last 20 trials of the staircases. These trials were then averaged within every staircase and subsequently averaged over staircases within each mask speed. Because of large inter-individual differences between thresholds, we normalized the thresholds by dividing them with the mean of the thresholds per participant. These normalized thresholds were then subjected to a Bayesian version of a one-way within-subjects ANOVA. Statistical inference throughout this paper did not use the classical frequentist framework but rather a Bayesian framework (see [46] for an introduction). Bayesian statistics offer a lot of advantages over the classical frequentist framework ([46]–[49], which has been disputed ever since it was introduced in psychology (e.g. from [50] to [47]). Moreover, using Bayesian inference as the principal way to do statistical inference is taken up more and more by researchers in vision science (e.g., see [51]).

In our analyses, we first do model selection using Bayes Factors. Subsequently, we use Bayesian parameter estimation to further zoom in on the posterior distributions. In both cases (model selection and parameter estimation) we have used the tools that are currently available. Note that these tools rely on different models with different, but in both cases uninformative, priors. Techniques for an integrated Bayesian approach to both model selection and parameter estimation are currently quite complicated to implement.

#### Bayesian model selection

**Figure 2:**
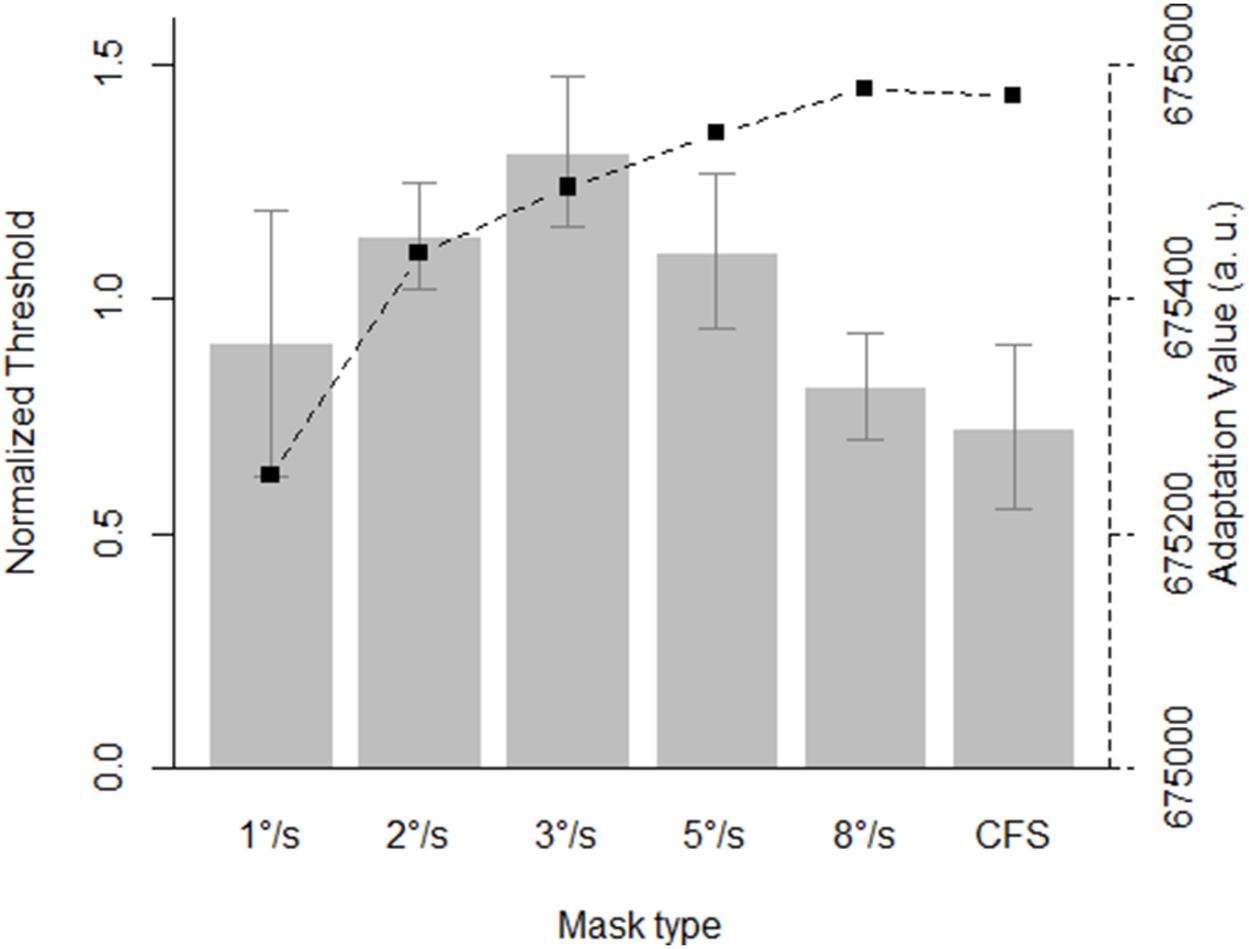
Mean normalized threshold in function of mask speed. The error bars denote 95% within-subject confidence intervals. The squares indicate simulated adaptation values (in arbitrary units) for each mask speed and regular CFS. Note that high values indicate *less* adaptation.

Rouder, Morey, Speckman, and Province [52] developed an approach in which a default class of priors is used to compute Bayes Factors in ANOVA designs. For an introduction, we refer to their paper. The Bayes Factor comparing a model with no effect and one with an effect of mask speed was equal to 13,992 indicating convincing evidence for a main effect of mask speed. Note that a classical repeated measures ANOVA yielded the same conclusion (*F*(5,20) = 6.205, *p = *.001). Figure 2 depicts the mean normalized threshold per condition and shows that the condition in which the speed of the CFS mask matched the speed of suppressed stimulus is the one with the highest threshold. In the next section, we will further zoom in on the results using Bayesian parameter estimation.

#### Bayesian parameter estimation

Figure S1 depicts the hierarchical model that was used to do Bayesian parameter estimation. This model was adapted from [46], [53]. The model is called hierarchical because it includes uncertainty at multiple levels. In contrast with classical repeated-measures ANOVA, the data were modeled as coming from a t-distribution instead of a normal to accommodate the possible influence of outliers. This method has also been called robust inference. Since the degrees of freedom of this t-distribution are unknown, it was treated as an unknown parameter and an uninformative uniform distribution was put on this parameter to let the data inform us about which degrees of freedom are in a credible range. The mean of the t-distribution is the result of a linear model (as in the classical repeated-measures ANOVA), comprising the general mean (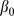), the effect of mask speed (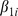) and the subjects factor (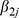). Furthermore, prior distributions are put on the parameters of the linear model. Note that these priors are not separate for each condition or subject, allowing that estimates for one condition inform estimates for the other or estimates for one subject are informed by estimates from other subjects. This is only one example of the flexibility of the Bayesian data-analytic approach and the advantage is that one has to be explicit about the assumptions included in the model that is used to analyze the data. Markov Chain Monte Carlo (MCMC) sampling was used to generate samples from the posterior distribution using the JAGS software.

Since the posterior distributions for 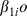 are deflections away from the baseline, contrasts can be computed to examine differences between two or more conditions – note that this is similar to performing a t-test. Here, we computed the difference between 3°/s and the average of all other conditions as well as pairwise comparisons between 3°/s and the other conditions. Figure S2 shows the posterior distributions associated with these contrasts. The black lines indicate the 95% highest density interval (HDI). This 95% HDI can be interpreted as an interval of credible parameter values. If this interval includes zero, we conclude that the compared conditions are not different and vice versa when zero falls out of the 95% HDI. Note that we can compute all these contrasts and do not have to use a correction for multiple comparisons. Indeed, there is just one (high-dimensional) posterior distribution and it does not change when you examine it in different ways ([46] pp. 284–285).

In summary, the data suggest that the normalized threshold for a mask speed of 3°/s is credibly different from the average threshold of all other mask speeds. Furthermore, pair-wise comparisons suggest that this difference holds for a mask speed of 1°/s and 8°/s. As a sanity check, Figure S3 depicts a posterior predictive check. In a posterior predictive check, every sample from the MCMC chain is used to predict a new data point by generating a random sample from the distribution you assume the data are generated from. If the model used for analyzing the data is not a good model, this would become clear from the predictions based on the believable parameter values. That is, these would deviate from the data or show a trend that is not present in the data. From Figure S3 it is apparent that the model used for this data set is a good model in the sense that it generates data that are in the range of the observed data.

#### Comparison with the simulations

In the Introduction, we suggested that the effectiveness of CFS does not entirely depend on retinotopically specific neural adaptation due to the continuous updates to the CFS mask. As the results of Experiment 1 indicate, the MMM that matched the motion properties of the suppressed target provided the most effective suppression. However, whilst it is logical to assume that the mask of 3°/s would show more retinotopically specific neural adaptation, it was important to quantify this explicitly, especially in relation to the traditional CFS mask. To address this, we computed a measure of the degree of retinotopic neural adaptation as described in the Methods section.

The squares in Figure 2 depict the results of the simulations. As is apparent from this figure, our implementation of retinotopically specific neural adaptation showed a continuous increase from the slowest to the fastest mask speed and regular CFS, where an increase indicates *less* adaptation (as explained in the Methods section). However, our results deviate from these simulations as an increase in thresholds up to 3°/s and a decrease in thresholds for masks with faster speeds was observed.

### Discussion

In Experiment 1, we manipulated the properties of MMMs and compared their effectiveness in suppressing a moving stimulus with regular CFS. We compared our pattern of results with that expected based on computations of the degree of retinotopic neural adaptation. If avoidance of retinotopically specific neural adaptation underlies the effectiveness of CFS, the mask with the most changes would prove to be the most effective. According to our measure of degree of retinotopic neural adaptation, the MMM and the regular CFS mask would show the least adaptation. However, the fastest mask speeds did not prove to be the most effective. It was apparent that the contrast threshold was highest for a MMM that matched the motion properties of the suppressed stimulus providing evidence for feature-selective depth of suppression during CFS (i.e., in line with [30], [37]).

Given the seemingly widespread assumption that effective CFS masking is driven by robustness to (retinotopic) neural adaptation, we tried to replicate our finding from Experiment 1 using two different to-be-suppressed target speeds. Thus, in Experiment 2 we manipulated the speed of the suppressed stimulus to move at either 2°/s or 5°/s whilst keeping the same range of mask speeds used as in Experiment 1. This also enables us to test the role of feature-selective depth of suppression during CFS. Indeed, analogous to the results of Yang and Blake [30] and compared to the results of Experiment 1, one would predict that the peak in the contrast threshold would shift toward a CFS mask where the speed is matched at 2°/s or 5°/s respectively for targets moving at 2°/s and 5°/s.

## Experiment 2

### Materials and Methods

#### Participants

Six new participants (1 male), all students of the undergraduate psychology program of the University of Leuven participated in the experiment for course credit. All had normal or corrected-to-normal vision. Every participant signed an informed consent prior to the start of the experiment and was naive to the goal of the study.

#### Apparatus and stimuli

The apparatus and stimuli were the same as in Experiment 1.

#### Design

Mask speed again consisted of six different levels (1°/s, 2°/s, 3°/s, 5°/s, 8°/s and regular CFS). Target speed was also manipulated and consisted of two levels (2°/s and 5°/s), yielding a 2×6 within-subjects design. Participants performed 65 trials for each staircase, resulting in 1,560 (65 trials×6 speeds×2 target speeds×2 staircases) trials in total. Target speed was blocked and counterbalanced across participants. In every block, staircases were randomly interleaved and participants had the opportunity to take small breaks in-between.

#### Procedure

The procedure of Experiment 2 was the same as in Experiment 1. The targets moved on a horizontal plane from the right side to the left and disappeared from the screen after 5.5 and 2.2 seconds, respectively for the 2°/s and 5°/s target speed conditions.

### Results

The data were analyzed in the same way as in Experiment 1. First, we report the results from Bayesian model selection and subsequently we elaborate on them using Bayesian parameter estimation.

#### Bayesian model selection

Bayes Factors were again computed based on Rouder et al. [52]. Different Bayes Factors are reported in Table 1, all of which can be interpreted as a comparison with a full model including the main effect of mask speed, the main effect of target speed and the interaction. Bayes Factors smaller than one indicate evidence for the full model.

**Table 1:** Bayes Factors associated with a comparison with the full model.

Model	Bayes Factor
**Null**	0.0003
**Mask Speed + Target Speed**	0.178
Target Speed + Mask Speed * Target Speed	0.0001
Mask Speed + Mask Speed * Target Speed	5.1146

**Figure 3:**
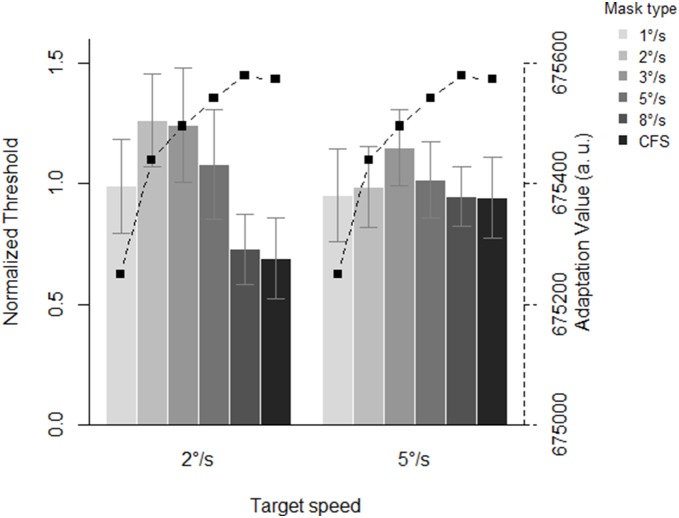
Mean normalized thresholds in function of target speed and mask speed. The error bars denote 95% within-subject confidence intervals. The squares indicate the simulated adaptation values (in arbitrary units) for each mask speed and regular CFS. Note that high values indicate *less* adaptation.

As the table shows, the model with a main effect of mask speed and an interaction between mask speed and target speed is strongly preferred. Note that a classical repeated measures ANOVA yields a similar conclusion (main effect of mask speed: *F*(5,25) = 4.066, *p* = .008; no main effect of target speed: *F*(1,5) = 1.346, *p* = .298; interaction between mask and target speed: *F*(5,25) = 2.156, *p* = .09). As is apparent from Figure 3 and in line with our predictions, the data indeed shift for the condition in which the target moved at 2°/s. The pattern of results is more complicated for the condition of 5°/s, however. Here, the thresholds seem to “flatten out” when the target moves at this speed.

#### Bayesian parameter estimation

Parameter estimation was done with a similar hierarchical model as in the analysis of Experiment 1, but with an extra main effect – target speed – added to the model. Figure S4 shows the associated graphical model. Because of the interaction, pair-wise comparisons were computed for every level of target speed. Figure S5 depicts the two pair-wise comparisons for a target speed of 2°/s that were credibly different from zero. In the 5°/s condition, no pair-wise comparisons were credibly different from zero.

### Discussion

As in Experiment 1, the results clearly deviate with those expected based on simulations of the degree of retinotopic neural adaptation for each condition. Experiment 1 also revealed a clear effect whereby the most effective mask was one in which the speeds were matched to those of the target. Using two new speeds in Experiment 2, we did find some additional evidence for the importance of the match between the speed of the mask and the stimulus, in that there was a significant interaction between the effectiveness of the different masking conditions across the two target speed conditions. Indeed for a target moving at 2°/s, the peak of the distribution of thresholds shifted more towards 2°/s compared to the results of Experiment 1. For a target moving at 5°/s, the results were less clear, in fact there were no credible differences between the masking speeds with a target moving at 5°/s. This is possibly due to the fact that the conditions were equated for distance covered over the display and not for presentation time. That is, the target stimuli crossed the same distance over the screen independent of the speed at which they moved, and thus stimuli in the 5°/s target condition are presented for a shorter time duration. It is possible therefore that, the shorter presentation time in the 5°/s condition renders it harder for the visual system to encode the speed of the target, and for this to then have any impact on the speeds used in the mask.

## General Discussion

In this study, we started from the observation that a MMM provided more effective suppression of a moving stimulus than a regular randomly updating CFS mask. This finding did not seem to readily follow from the current assumptions regarding why CFS is an effective suppression paradigm. The robust nature of CFS suppression is generally considered to be the result of the transient nature of the mask, reducing the amount neural adaptation during the interocular competition process at retinotopic stages of the visual system (which have often been implicated in the competition process [31], [32]). The continuously moving masks we employed yielded a higher degree of simulated retinotopic neural adaptation than the regular CFS mask and therefore should have been less effective (particularly for the slower motion speeds).

In Experiment 1, the speed of individual mask elements of the MMM was manipulated. The influence of varying this speed on masking a moving stimulus was tested and compared to traditional CFS. The data showed an effect of mask speed on the contrast thresholds at which the target could be detected. The highest threshold was obtained for the mask speed that matched the speed of 3°/s at which the target stimulus moved. The thresholds decreased as the CFS mask moved either slower or faster. This finding highlights that regular CFS is not always a powerful, readily applied interocular suppression technique. Instead, the findings of Experiment 1 highlight the feature-selective depth of interocular suppression through CFS. That is, when the properties of the mask are more similar to the suppressed stimulus, suppression is stronger (see [30] for a further discussion of feature-selective depth of suppression).

Secondly, we explicitly contrasted our findings with a simulation of the degree of retinotopically specific neural adaptation. If transients were critical for CFS to be effective, the mask that contained most feature changes over time was expected to be the most effective. According to the adaptation measure we computed, the masks that showed the least adaptation were the regular CFS, and the moving masks with the highest speeds. However, as shown in Figure 2, these were not observed to be the most effective with respect to suppressing the target stimulus ruling out an explanation of the effectiveness of CFS in terms of a simple approximation of retinotopic neural adaptation.

In Experiment 2, we manipulated the speed of the target stimulus and found that the distribution of thresholds changed when the speed of the target stimulus was changed. The results from the second experiment provided some additional support for the importance of matching between the stimulus and the mask, though this interaction was not clear in the 5°/s condition. More critically to our current goal, the results from Experiment 2 again did not agree with the predictions of what would be the most effective mask based on our simulations of retinotopic adaptation.

Although the predictions derived from the simulation of retinotopic adaptation did not agree with the data obtained in both experiments, we should note explicitly that we are not claiming that we have derived predictions from a complete or full-blown model of retinotopic adaptation. Our implementation aimed specifically at extracting edges at different locations in our CFS images and then applying an adaptation process depending on the orientation and contrast polarity of these edges, akin to what a primary visual cortex complex cell might be doing. Thus, in our simulations we did not consider varying spatial frequencies nor differential response properties for magno- and parvocellular pathways. Further, with respect to the adaptation process, we only used one timescale for adaptation and one for recovery (based on neurophysiological measurements), whereas adaptation on multiple timescales or different timescales for different features might be possible. Our simulation, therefore, should only be interpreted as a coarse approximation of retinotopic adaptation. Yet we think this operationalization captures the essence of the concept of retinotopic adaptation which has been proposed as being important for the effectiveness of CFS.

Methodologically, our results highlight that one should consider using MMM instead of a traditional CFS mask in some contexts to achieve desirable suppression strength. Indeed, our most consistent finding was that a MMM, and especially one that contained motion features similar to the suppressed stimulus was more effective than a traditional CFS mask, highlighting the importance of binocular feature matching [30], [37]. Thus, our results suggest that researchers wanting to suppress moving stimuli should also focus on developing MMMs.

Theoretically, our results highlight that the transient nature of the mask is not always the most important aspect of CFS, in the sense that the more spatially transient the mask is, the more effective suppression will be. The initial innovation in developing CFS was exactly the introduction of a transient in one eye which indeed seems crucial for the increase in suppression strength [29]. However, the relationship between mask transients and effective suppression does not seem to be as simple as one might assume based on retinotopically specific neural adaptation. Indeed, to achieve reliable suppression through CFS one has to consider the feature similarity between mask and target. This reconnects our understanding of CFS with observations from the binocular rivalry literature in which the importance of feature similarity of competing stimuli has repeatedly been shown [38]–[40].

Whilst the current results challenge the idea that the effectiveness of CFS can be predicted based on a reduction in retinotopically specific neural adaptation, they do not imply that no adaptation-based processes underlie the effect. Indeed, these could potentially be explained by an adaptation mechanism acting at the level of motion speed, for example. That is, given that the visual system can adapt to motion speed [54], [55], the condition in which mask speed and target speed overlapped would increase the level of adaptation to that specific speed and potentially increase the thresholds for the detection of that speed consistent with our results.

Alternatively, one could also speculate that a mask moving at 3°/s would activate parts of motion area MT that also would be required to represent the target moving at 3°/s. This explanation would be more consistent with the idea that interocular competition results from a bottleneck imposed by the selective access to higher level areas. If the target and mask in CFS share more properties, then it is possible that they compete more directly for the same neural resources. The stronger motion signals in the mask could dictate that only the mask stimulus reaches higher areas and therefore stays dominant and increases detection thresholds for the suppressed stimulus.

This second explanation could potentially be related to a broader mechanism implicated in the singleton pop-out literature using visual search. In this literature, target-nontarget similarity has been shown to have an influence on the slope of the search function [56] such that the slope is observed to be higher as the similarity between target and nontarget increases. Indeed, in our experiments we observed that it was increasingly easier for participants to detect the moving stimulus when the similarity between mask and target stimulus decreased.

As is apparent from our experiments, and consistent with the work of Hong and Blake [36], Maehara et al. [37], and Yang and Blake [30], the specific properties of the mask play an important role. Indeed, this is also reflected in a recent attempt to construct a dynamical systems model of CFS [26]. This model, which extends a minimal model for binocular rivalry introduced by [57], includes a feature-selective component in addition to the classical cross-inhibition and self-adaptation components.

Thus, regular CFS does not seem to be a general panacea for suppressing stimuli. Indeed, one has to take into account the similarity between features that can be extracted based on the input to each eye, rather than simply increasing the transients in the mask. This finding could help to account for the (implicit) tendency in the literature for different authors to adapt the CFS mask based on the stimulus they are trying to suppress, presumably by matching more closely the characteristics of the to-be-suppressed stimuli and the mask.

## Conclusion

In this study, we introduced a MMM that was shown to be more effective in suppressing a moving stimulus than a regular CFS mask. We developed an explicit quantification of the degree of retinotopically specific neural adaptation and used this to make predictions on the effectiveness of our masks. Our results were not consistent with the predictions based on the approximation of retinotopic neural adaptation, and this questions the assumption that the most effective mask will always reflect the avoidance of neural adaptation due to the transient nature of the CFS mask. We conclude that a regular CFS mask that provides effective suppression for static stimuli is not necessarily suited for suppressing moving stimuli and that in general one has to consider the feature match between mask and suppressed stimulus when attempting to use CFS.

## Supporting Information

Figure S1
**Graphical model for the Bayesian version of a one-way repeated measures ANOVA.** The data are assumed to come from a t-distribution with a certain mean and standard deviation. The mean is equal to a linear combination of the effect of mask speed (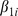) and a participant-specific effect (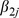).(TIF)

Figure S2 **Posterior distributions for the pair-wise comparisons between 3°/s and all other levels of mask speed and the regular CFS mask.**
(TIF)

Figure S3 **Posterior predictive checks for every participant.** The red cross is the mean of the predicted values, the gray line the associated 95% HDI and the black dots are the individual data points for every condition. The conditions are ordered as in the bar plots going from a mask moving at 1°/s to regular CFS.
(TIF)

Figure S4
**Graphical model for the Bayesian version of a two-way repeated measures ANOVA.** The data are assumed to come from a distribution with a certain mean and standard deviation. The mean is equal to a linear combination of the effect of mask speed (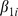), target speed (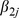), their interaction (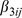) and a participant-specific effect (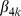).(TIF)

Figure S5 **Posterior distributions for the pair-wise comparisons between a mask moving at 2°/s and 8°/s and 2°/s and CFS, respectively, for the 2°/s target speed condition.**
(TIF)
