# Supplementary figures and images for: Moving Stimuli Are Less Effectively Masked Using Traditional Continuous Flash Suppression (CFS) Compared to a Moving Mondrian Mask (MMM): A Test Case for Feature-Selective Suppression and Retinotopic Adaptation
Source: PLoS One. 2014 May 30;9(5):e98298. doi: 10.1371/journal.pone.0098298 (PMC4039473; doi:10.1371/journal.pone.0098298)

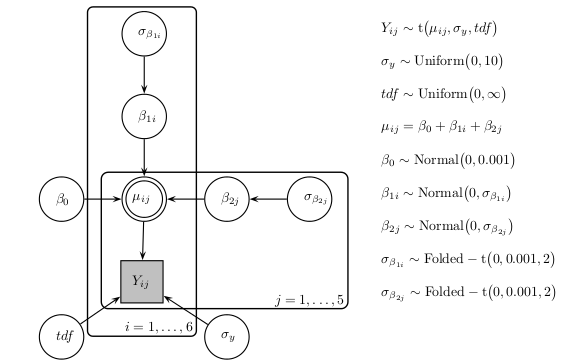

Supplement: Figure S1 — Graphical model for the Bayesian version of a one-way repeated measures ANOVA. The data are assumed to come from a t-distribution with a certain mean and standard deviation. The mean is equal to a linear combination of the effect of mask speed () and a participant-specific effect (). (TIF) [file pone.0098298.s001.tif]

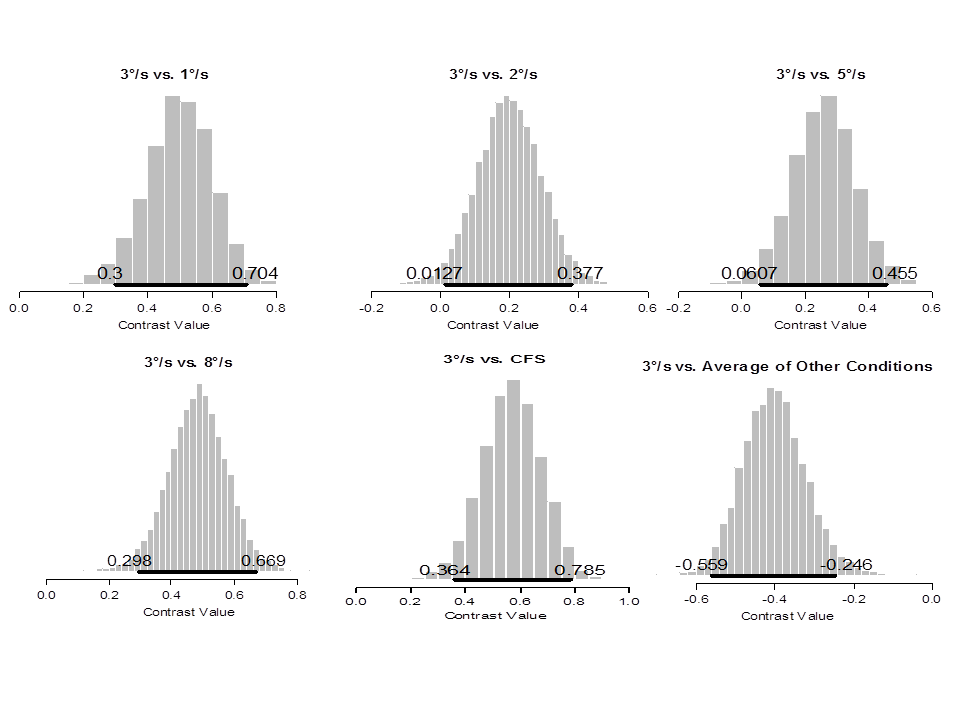

Supplement: Figure S2 — Posterior distributions for the pair-wise comparisons between 3°/s and all other levels of mask speed and the regular CFS mask. (TIF) [file pone.0098298.s002.tif]

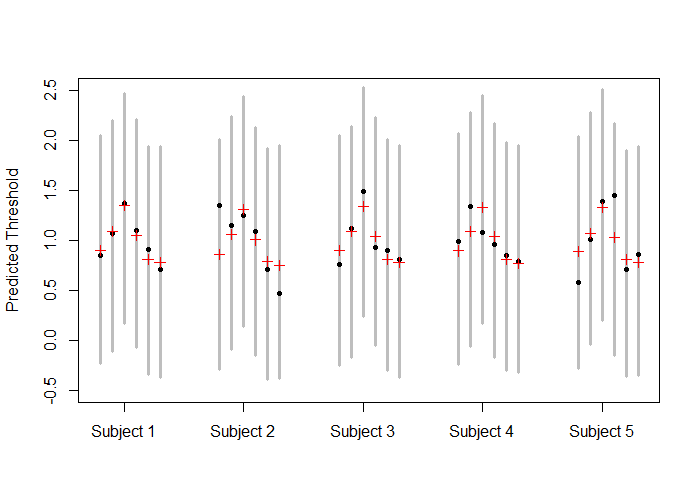

Supplement: Figure S3 — Posterior predictive checks for every participant. The red cross is the mean of the predicted values, the gray line the associated 95% HDI and the black dots are the individual data points for every condition. The conditions are ordered as in the bar plots going from a mask moving at 1°/s to regular CFS. (TIF) [file pone.0098298.s003.tif]

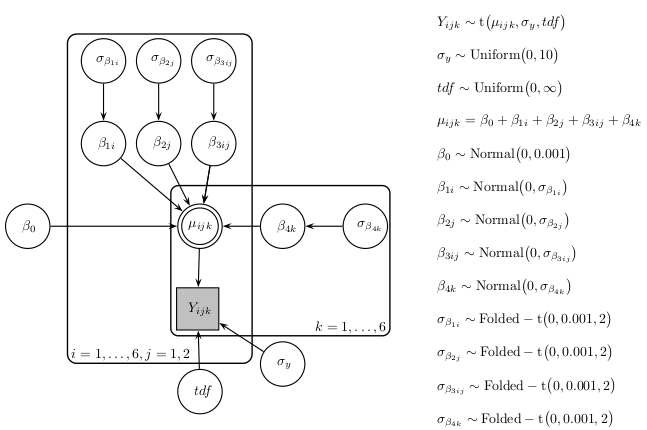

Supplement: Figure S4 — Graphical model for the Bayesian version of a two-way repeated measures ANOVA. The data are assumed to come from a distribution with a certain mean and standard deviation. The mean is equal to a linear combination of the effect of mask speed (), target speed (), their interaction () and a participant-specific effect (). (TIF) [file pone.0098298.s004.tif]

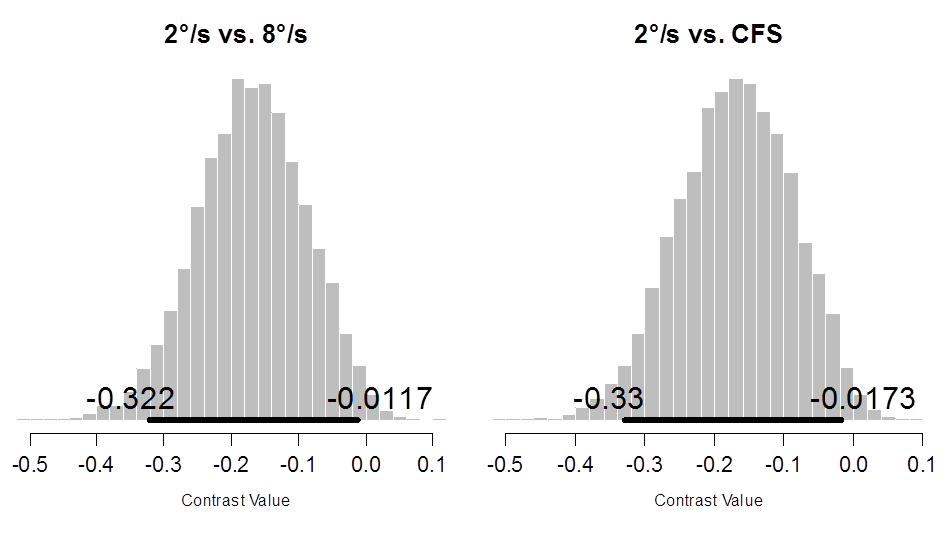

Supplement: Figure S5 — Posterior distributions for the pair-wise comparisons between a mask moving at 2°/s and 8°/s and 2°/s and CFS, respectively, for the 2°/s target speed condition. (TIF) [file pone.0098298.s005.tif]
